# The predictive ability of occipital to C3 angle for dysphagia after occipitocervical fusion in patients with combined C2–3 Klippel-Feil syndrome

**DOI:** 10.1186/s12891-022-05072-8

**Published:** 2022-02-07

**Authors:** Qiang Zou, Linnan Wang, Xi Yang, Yueming Song, Limin Liu, Lei Wang, Zhongjie Zhou, Bowen Hu, Taiyong Chen, Hao Liu

**Affiliations:** Department of Orthopedics, |Orthopedic Research Institute, West China Hospital, Sichuan University, No. 37 Guo Xue Xiang, Chengdu, 610041 Sichuan China

**Keywords:** Occipitocervical fusion, Klippel-Feil syndrome, Dysphagia, Occipital-C3 angle

## Abstract

**Background:**

Improper occipitocervical alignment after occipitocervical fusion (OCF) may lead to devastating complications, such as dysphagia and/or dyspnea. The occipital to C2 angle (O-C2a), occipital and external acoustic meatus to axis angle (O-EAa) have been used to evaluate occipitospinal alignment. However, it may be difficult to identify the inferior endplate of the C2 vertebra in patients with C2–3 Klippel-Feil syndrome (KFS). The purpose of this study aimed to compare four different parameters for predicting dysphagia after OCF in patients with C2–3 KFS.

**Methods:**

There were 40 patients with C2–3 KFS undergoing OCF between 2010 and 2019. Radiographs of these patients were collected to measure the occipital to C3 angle (O-C3a), O-C2a, occipito-odontoid angle (O-Da), occipital to axial angle (Oc-Axa), and narrowest oropharyngeal airway space (nPAS). The presence of dysphagia was defined as the patient complaining of difficulty or excess endeavor to swallow. Patients were divided into two groups according to whether they had postoperative dysphagia. We evaluated the relationship between each of the angle parameters and nPAS and analyzed their influence to the postoperative dysphagia.

**Results:**

The incidence of dysphagia after OCF was 25% in patients with C2–3 KFS. The Oc-Axa, and nPAS were smaller in the dysphagia group compared to non-dysphagia group at the final follow-up (*p* < 0.05). Receiver-operating characteristic (ROC) curves showed that dO-C3a had the highest accuracy as a predictor of the dysphagia with an area under the curve (AUC) of 0.868. The differences in O-C3a, O-C2a, O-Da, and Oc-Axa were all linearly correlated with nPAS scores preoperatively and at the final follow-up within C2–3 KFS patients, while there was a higher R^2^ value between the dO-C3a and dnPAS. Multiple linear regression analysis showed that the difference of O-C3a was the only significant predictor for dnPAS (β = 0.670, *p* < 0.001).

**Conclusions:**

The change of O-C3a (dO-C3a) is the most reliable indicator for evaluating occipitocervical alignment and predicting postoperative dysphagia in C2–3 KFS patients. Moreover, dO-C3a should be more than − 2° during OCF to reduce the occurrence of postoperative dysphagia.

## Background

Occipitocervical fusion (OCF) was first reported by Foerster in 1927, attributed to the continuous improvement and advancement of this technology and has been increasingly performed in upper cervical spinal lesions, such as injuries, tumors, rheumatism, congenital deformities and degenerative processes [1–5]. Although OCF can reconstruct upper cervical alignment and provide an excellent fusion rate, complications after OCF, including hematoma, infection, respiratory disorders, and dysphagia, are not uncommon [6–8]. Dysphagia has been recognized as a catastrophic complication after OCF, with an incidence ranging from 15.8 to 26.6% [3, 9–11]. This situation may sacrifice the quality of life of patients and sometimes poses a serious threat to their daily life [7, 8, 12]. Therefore, to prevent postoperative dysphagia, clinicians should pay sufficient attention to performing OCF surgery before fixing screws.

Some scholars have shown that patients’ complaints of dysphagia were self-reported [3, 6, 10, 11, 13, 14]. Besides, past studies have discussed the possible underlying mechanism of dysphagia and dyspnea after OCF is mainly mechanical stenosis of the airway space [6, 8, 14]. Some researchers have revealed a correlation between a reduction in the occipital to C2 angle (O-C2a) and a decrease in the pharyngeal airway space. They thought that a reduction in the O-C2a was considered a risk factor for dysphagia after OCF [8, 14, 15]. Therefore, the difference between the postoperative and preoperative O-C2a (dO-C2a) was used to predict postoperative dysphagia [13]. Meng et al. [3] showed that clinicians should ensure a dO-C2a greater than − 5° to effectively avoid postoperative dysphagia and correct the O-C2a just before the final occipitocervical fixation, if the dO-C2a during surgery is less than − 5°. Later, Morizane et al. [16] proposed the occipital and external acoustic meatus to axis angle (O-EAa) to indicate craniocervical junction alignment, which could reflect the translation of the cranium in relation to C2 and may affect the narrowest oropharyngeal airway space (nPAS). Chen et al. [10] suggested that maintaining a postoperative O-EAa value of 100° would reduce postoperative dysphagia. Therefore, maintenance of the O-C2a and O-EAa at an appropriate level is a practical and effective method to prevent postoperative dysphagia.

**Fig. 1:**
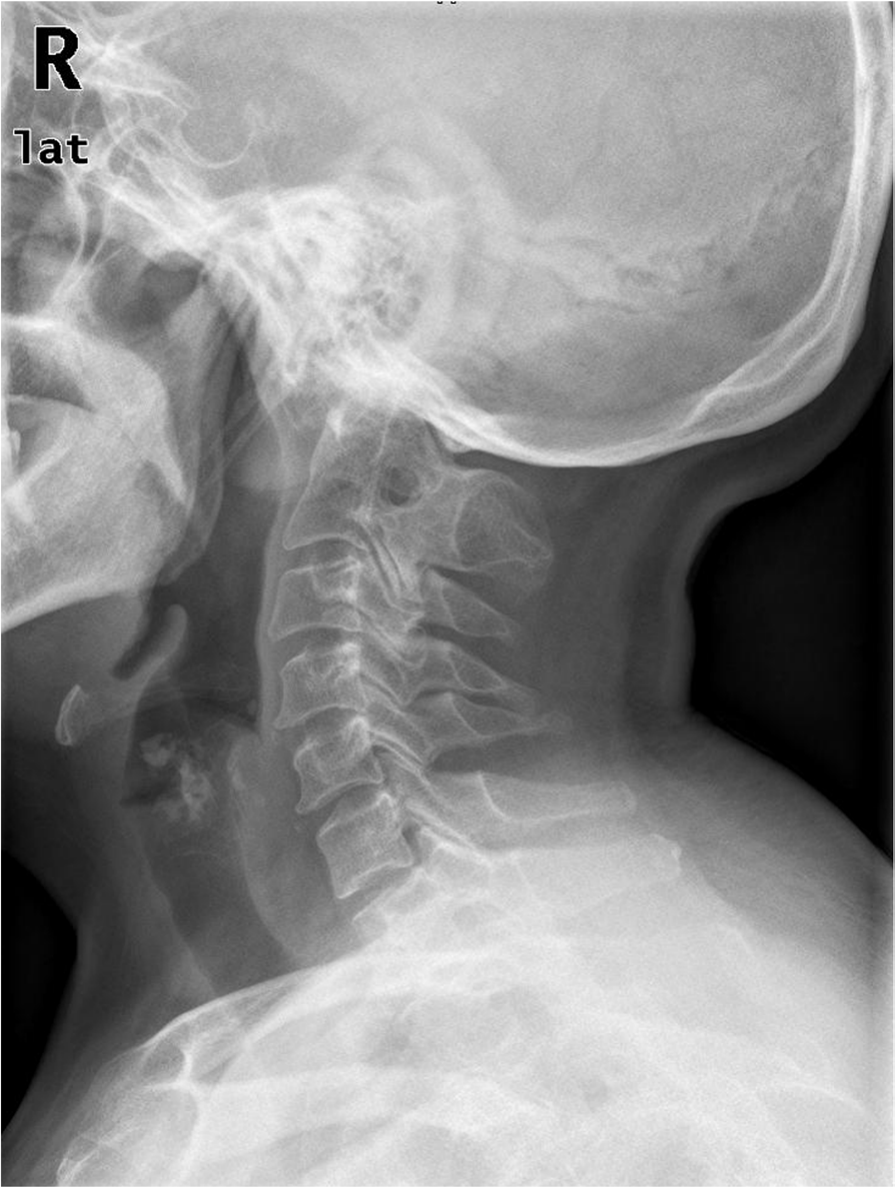
64-year-old woman, atlantoaxial dislocation, basilar invagination. Inferior endplate of the C2 vertebra was unclear due to C2–3 fusion

Klippel-Feil syndrome (KFS) is a rare congenital malformation featuring fusion of 2 or more cervical vertebrae**.** Previous studies have revealed that the incidence of KFS is estimated to be approximately 1 in 40,000 to 42,000 newborns [17, 18]. Gruber et al. [19] reported that the most common lesion was an isolated fusion of the second and third cervical vertebrae (C2–3). However, there may be some measuring error in identifying the inferior endplate of the C2 vertebra by O-C2a and O-EAa in KFS patients with C2–3 vertebral fusion (Fig. 1). Therefore, Nagashima et al. [20] recommended using the occipital to axial angle (Oc-Axa) as a reliable indicator for assessing occipitocervical alignment using intraoperative fluoroscopic imaging, which is particularly useful in cases where it is difficult to identify the inferior endplate of the C2 vertebra. However, they did not propose the exact value of the Oc-Axa to predict dysphagia. In 2020, Bellabarba et al. [21] proposed the mandible-C2 angle to assess occipitocervical alignment, which may be appropriate for trauma patients in whom a baseline preoperative reference point is not available. However, they evaluated radiographs of patients who had no fracture, malalignment or any other pathologic finding.

Until now, how to measure the occipitocervical angle more precisely in patients with unclear inferior C2 endplates (such as in patients with C2–3 KFS) has not been reported in the literature. The purpose of this study was to compare the ability of four different measurement parameters for predicting dysphagia after OCF in patients with C2–3 KFS.

## Methods

### Patient population

This study was approved by the Ethics Board of the local institution, and all patients provided written informed consent to participate in the study. A total of 40 consecutive patients with C2–3 KFS (17 males and 23 females) who underwent OCF surgery for the treatment of craniocervical junction instability from April 2010 to August 2019 were included in this study. The inclusion criteria were the diagnosis of C2–3 KFS, undergoing OCF surgery, a minimum follow-up of 1 year, and a complete preoperative and postoperative radiographic record. The exclusion criteria were as follows: (1) preoperative dysphagia, (2) previous transoral surgery, and (3) unavailable data for evaluating postoperative swallowing conditions.

### Radiographic measurement

**Fig. 2:**
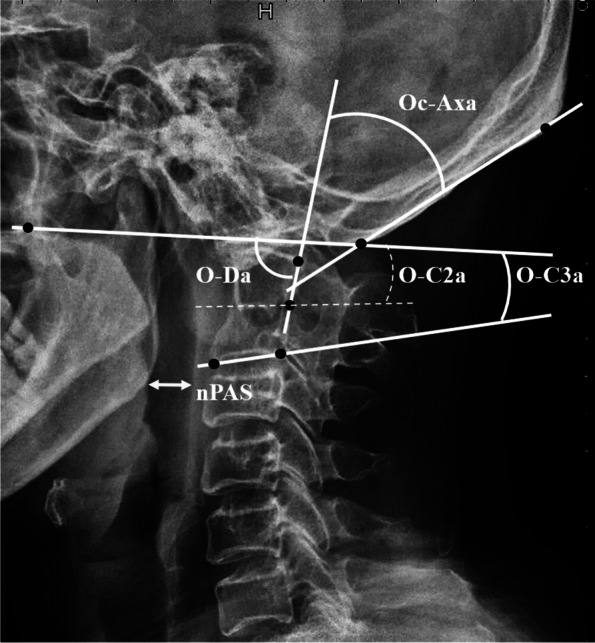
Representation of radiographic measurements. The O-C3a was defined as the angle between the McGregor line and the inferior endplate of C3. The O-C2a represented the angle between the McGregor line and the inferior endplate of C2. The occipito-odontoid angle (O-Da) was formed by the angle between the McGregor line and the posterior longitudinal line of the C2 vertebra. The Oc-Axa was defined as the angle between the line connecting the occipital protuberance and the most caudal point on the midline occipital curve and the posterior longitudinal line of the C2 vertebra. The nPAS was defined as the narrowest anterior-posterior diameter of the oropharynx, which was measured at the level between the tips of the uvula and epiglottis

Two senior spinal surgeons measured the following craniocervical radiological measurement parameters on lateral cervical radiographs taken before surgery and at the final follow-up: O-C3a, O-C2a, O-Da, Oc-Axa, and nPAS (Fig. 2). The O-C3a was defined as the angle between the McGregor line and the inferior endplate of C3. The O-C2a represented the angle between the McGregor line and the inferior endplate of C2. The occipito-odontoid angle (O-Da) was formed by the angle between the McGregor line and the posterior longitudinal line of the C2 vertebra. The Oc-Axa was defined as the angle between the line connecting the occipital protuberance and the most caudal point on the midline occipital curve and the posterior longitudinal line of the C2 vertebra. The nPAS was defined as the narrowest anterior-posterior diameter of the oropharynx, which was measured at the level between the tips of the uvula and epiglottis.

Radiological measurement parameters were taken twice on different days (at least a 14-day interval) by two senior spinal surgeons. The value of the parameter is the average of the two measurements.

### Dysphagia assessment

After reviewing the medical records of all the patients, there was no dysphagia before OCF surgery, and swallowing ability was evaluated at each outpatient follow-up visit or telephone interview. The presence of dysphagia is defined as the patient complaining of difficulty or excess endeavor to swallow [22]. Dysphagia grading system created by Bazaz was used for this study. The patients with no episodes of swallowing difficulty were graded as “none.” The patients who suffered rare episodes of dysphagia were graded as “mild.” Suffering occasional swallowing difficult with specific foods was classified as “moderate.” Severe dysphagia was defined as frequent difficult swallowing with the majority of foods [23].

### Statistical analysis

SPSS 22.0 software (SPSS Inc., Chicago, IL) was used to analyze the collected data. Continuous variables were expressed as means and standard deviations. Categorical variables were presented as numbers. Continuous variables were compared using Student’s t test or the Mann-Whitney U test. Categorical variables were compared using the chi-square or Fisher exact test. Receiver operating characteristic (ROC) curves for dO-C3a, dO-C2a, dO-Da, dOc-Axa (difference between final follow-up and preoperative O-C3a, O-C2a, O-Da, and Oc-Axa) were constructed and the areas under the curve (AUC) were calculated to identify and compare the accuracy of these parameters in predicting postoperative dysphagia. A parameter was considered as high accuracy when the AUC is > 0.9, while 0.7 to 0.9 indicates moderate accuracy [24]. The optimal cutoff point of these angle parameters for predicting postoperative dysphagia were determined by ROC curves using the Youden index (J) [25, 26]. A simple linear regression was used to investigate the association of the dO-C3a, dO-C2a, dO-Da, and dOc-Axa with dnPAS. In addition, multiple regression analysis was used to examine the influencing factors of dnPAS. Statistical significance was defined as *p* < 0.05.

## Results

The incidence of dysphagia in patients with C2–3 KFS after OCF was 25% (10/40). 6 patients were diagnosed as basilar invagination (BI), 4 atlantoaxial subluxation (AS), 26 BI with AS, 3 rheumatoid arthritis (RA) with AS, 1 malunion of odontoid fracture with AS. According to Bazaz’ dysphagia classification, mild dysphagia was present in 5 patients, moderate in 4 patients, and severe in 1 patient after surgery. This swallowing difficulty was mild in 8 patients and moderate in 2 patients at final follow-up assessment. All patients suffering post-operative dysphagia were reluctant to undergo further revision surgery because improvement in neurological function was acceptable. When the patients were split into dysphagia and non-dysphagia groups, there was no intergroup significant difference in terms of the average follow-up time, gender, age, fixed segments (separated by ≤C3 and > C3), proportion of patients with RA, or proportion of patients with anterior release surgery (ARS). Table 1 enumerates the demographic data of the patients.

**Table 1:** Patients’ demographic data (Mean ± SD)

	Dysphagia	Without dysphagia	*p* Value
Number of cases	10	30	
Gender (Male:Female)	4:6	13∶17	1.000
Mean age (y)	57.2 ± 9.5	52.7 ± 14.5	0.366
Follow-up (mo)	77.4 ± 36.6	70.2 ± 29.8	0. 537
BMI (Kg/m^2^)	23.1 ± 3.5	24.2 ± 7.9	0.668
Fixed segments (≤C3: >C3)	5:5	18:12	0.853
RA: without RA	2:8	1:29	0.149
ARS: without ARS	0:10	2:28	1.000

*SD* Standard deviation, *BMI* Body Mass Index, *RA* Rheumatoid arthritis, *ARS* Anterior release surgery

* *p* < 0.05

There was no significant difference between preoperative and final follow-up for the O-C3a (preoperative: 8.1 ± 9.2° vs. final follow-up: 8.4 ± 8.0°, *p* = 0.823), O-C2a (preoperative: 5.4 ± 12.7° vs. final follow-up: 5.8 ± 10.9°, *p* = 0.979), O-Da (preoperative: 83.6 ± 12.7° vs. final follow-up: 83.3 ± 10.7°, *p* = 0.826), Oc-Axa (preoperative: 58.7 ± 15.4° vs. final follow-up: 60.2 ± 12.2°, *p* = 0.491), and nPAS (preoperative: 1.4 ± 0.5 cm vs. final follow-up: 1.4 ± 0.5 cm, *p* = 0.499).

After patients were divided into two groups according to whether they had developed postoperative dysphagia, we found that the preoperative O-C3a, O-C2a, O-Da, Oc-Axa, and nPAS values were not significantly different between the groups (*p* > 0.05). The values of O-C3a, O-C2a, O-Da at final follow-up did not show significant difference between the groups (*p* > 0.05). The Oc-Axa, and nPAS were smaller in the dysphagia group compared to those in the nondysphagia group at the final follow-up (*p* < 0.05). The variations in the O-C3a (dO-C3a: − 5.4 ± 3.7°° vs. 2.2 ± 7.3°, *p* = 0.003), O-C2a (dO-C2a: − 6.0 ± 7.0°° vs. 2.6 ± 8.2°, p = 0.001), O-Da (dO-Da: − 7.7 ± 7.5° vs. 2.2 ± 6.5°, *p* < 0.001), Oc-Axa (dOc-Axa: − 4.2 ± 6.41° vs. 2.5 ± 7.1°, *p* = 0.011), and nPAS (dnPAS: − 0.4 ± 0.3 cm vs. 0.1 ± 0.5 cm, *p* = 0.003) were distinctly different between the groups. Table 2 shows and analyzes the preoperative and final follow-up radiographic parameters of the two groups.

**Table 2:** Patients’ radiographic outcomes (mean ± SD)

	Dysphagia	Without dysphagia	*p* Value
Pre- O-C3a	10.1 ± 8.2	7.5 ± 9.5	0.431
Pre- O-C2a	8.5 ± 9.3	4.4 ± 13.6	0.389
Pre-O-Da	87.3 ± 10.7	82.4 ± 13.2	0.300
Pre-Oc-Axa	54.5 ± 13.6	56.2 ± 12.8	0.716
Pre-nPAS	1.2 ± 0.6	1.5 ± 0.5	0.220
Final-O-C3a	4.7 ± 5.5	9.6 ± 8.4	0.095
Final-O-C2a	2.5 ± 9.1	7.0 ± 11.3	0.264
Final -O-Da	79.6 ± 8.2	84.6 ± 11.2	0.196
Final -Oc-Axa	50.2 ± 9.3	58.7 ± 10.1	0.025*
Final-nPAS	0.8 ± 0.3	1.5 ± 0.4	<0.001*
dO-C3a	−5.4 ± 3.7	2.2 ± 7.3	0.003*
dO-C2a	−6.0 ± 7.0	2.6 ± 8.2	0.001*
dO-Da	−7.7 ± 7.5	2.2 ± 6.5	<0.001*
dOc-Axa	−4.2 ± 6.4	2.5 ± 7.1	0.011*
dnPAS	−0.4 ± 0.3	0.1 ± 0.5	0.003*

*SD* Standard deviation, *Pre*- Preoperative, *Final-* Final follow-up, dO-C3a, dO-C2a, dO-Da and dOc-Axa, the difference between final follow-up and preoperative O-C3a, O-C2a, O-Da and Oc-Axa; dnPAS, the difference between final follow-up and preoperative minimum width of the oropharyngeal airway space

* *p* < 0.05

**Fig. 3:**
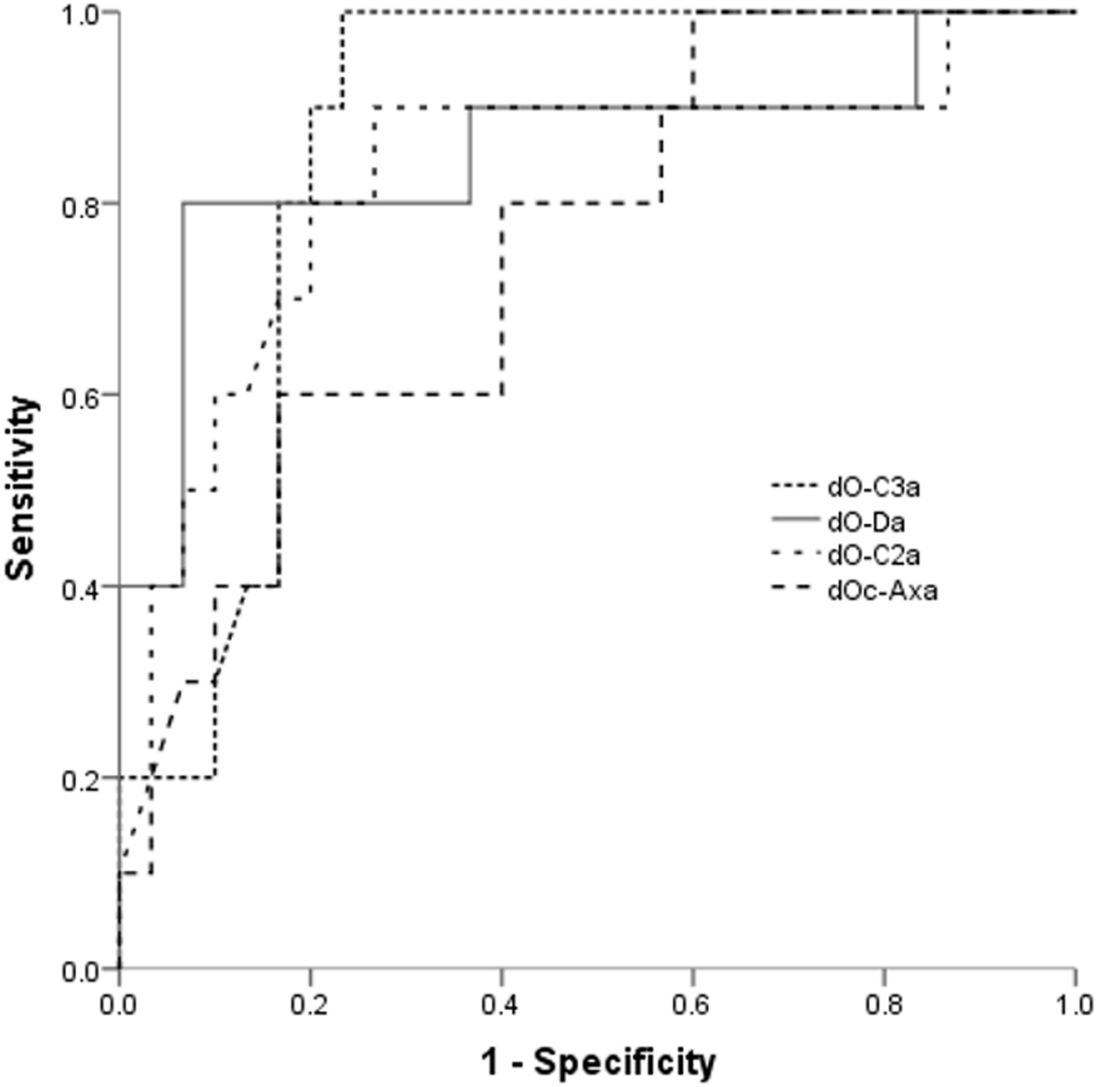
Receiver operating characteristic (ROC) curves of different angle parameters for predicting the occurrence of postoperative dysphagia

The ROC curves showed that dO-C3a, dO-C2a, dO-Da, and dOc-Axa had moderate accuracy as predictors for postoperative dysphagia in C2–3 KFS patients undergoing OCF surgery with an AUC of 0.868 [95% confidence interval (CI): 0.758–0.978], 0.827 (95% CI: 0.659–0.995),0.853 (95% CI: 0.688–1.000), and 0.752 (95% CI: 0.587–0.916) respectively (Fig. 3). The optimal cutoff points of dO-C3a, dO-C2a, dO-Da, and dOc-Axa were − 2°, − 2.2°, − 6°, and − 4.4° respectively.

**Fig. 4:**
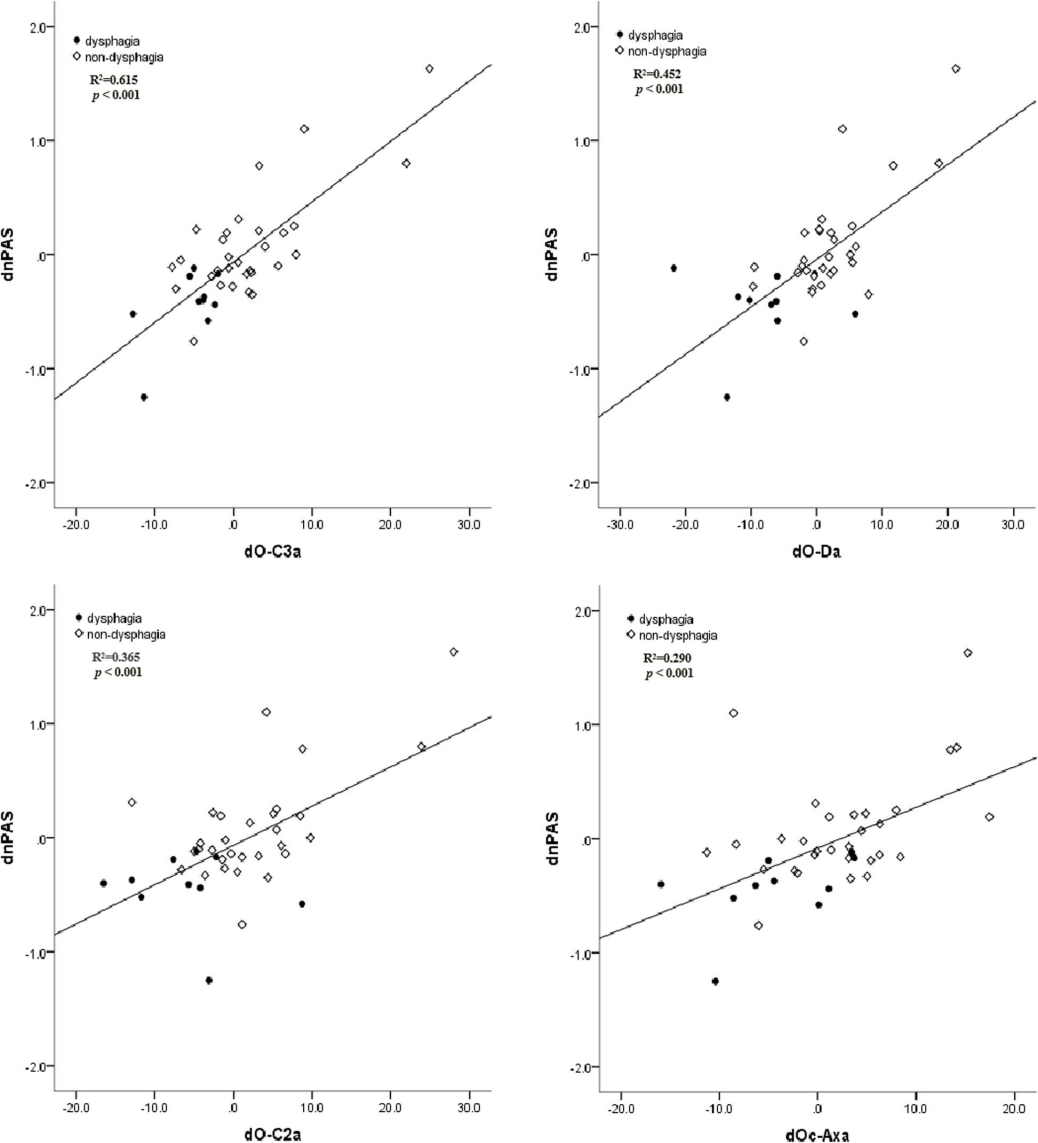
Scatter diagram showing the linear relationship between dO-C3a, dO-Da, dO-C2a, dOc-Axa and dnPAS

The change between the O-C3a at the preoperative and final follow-up is linearly correlated with nPAS in patients with C2–3 KFS (R^2^ = 0.615, *p* < 0.001), as shown in Fig. 4. The scatter diagram showed a linear correlation of dO-C2a and dO-Da with dnPAS (R^2^ = 0.365, *p* < 0.001; R^2^ = 0.452, *p* < 0.001), and there was a linear relationship between the dOc-Axa and dnPAS (R^2^ = 0.290, *p* < 0.001). The R^2^ value of the correlation between the dO-C3a and dnPAS was greater than dO-C2a, dO-Da and dOc-Axa (Fig. 4). In addition, multiple regression analysis showed a significant correlation between dO-C3a and dnPAS (dO-C3a: β = 0.670, p < 0.001) (Table 3).

**Table 3:** Multivariate linear regression analysis for the influence of predictors on dnPAS

Predictors for dnPAS	Unstandardized coefficients			95.0% Confidence interval			
	B	SE	β	Lower limit	Upper limit	t Value	*p*
dO-Da	0.017	0.009	0.276	−0.002	0.036	1.821	0.077
dO-C3a	0.045	0.012	0.670	0.022	0.069	3.899	<0.001*
dO-C2a	−0.012	0.011	− 0.214	−0.034	0.009	−1.163	0.253
dOc-Axa	0.009	0.009	0.142	−0.009	0.027	1.061	0.296
(Constant)	−0.063	0.049		−0.162	0.036	−1.288	0.206

Adjusted R^2^ = 0619, *P* < 0.001

dO-C3a, dO-C2a, dO-Da and dOc-Axa, the difference between final follow-up and preoperative O-C3a, O-C2a, O-Da and Oc-Axa; dnPAS, the difference between final follow-up and preoperative minimum width of the oropharyngeal airway space

**Fig. 5:**
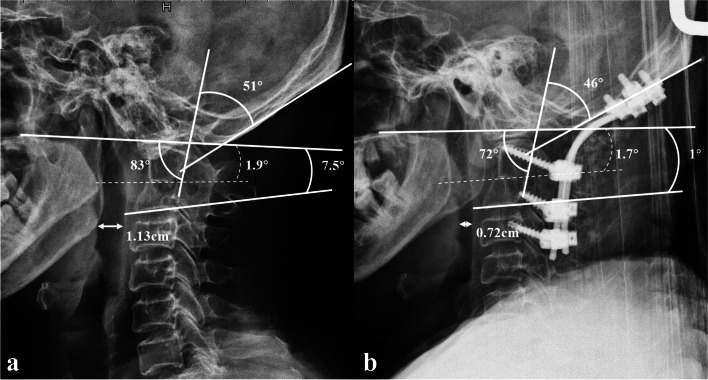
Lateral radiograms of a patient suffering dysphagia. a. Preoperative values of O-C3a, O-C2a, O-Da, Oc-Axa and nPAS were 7.5°,1.9°, 83°, 51°, and 1.13 cm respectively. b. Postoperative O-C3a, O-C2a, O-Da, Oc-Axa and nPAS decreased to 1°, 1.7°,72°, 46°, and 0.72 cm respectively

Lateral radiograms of a patient suffering dysphagia are shown in Fig. 5.

## Discussion

Previous studies have suggested that the mechanism of dysphagia after occipitocervical fusion may be caused by oropharyngeal airway stenosis. The following causes may lead to oropharyngeal stenosis: anatomical abnormalities due to RA, postoperative hematoma, edema of the airway due to intubation, anterior decompression of anterior atlantoaxial subluxation, and improper fixation angle of the upper cervical vertebra [3, 8, 13, 15, 27]. Extensive investigations measuring cervical lateral radiographs before and after surgery found that oropharyngeal stenosis was closely related to the reduced occipitocervical angle after fixation, and some specific occipitocervical angles could be used as imaging indicators to predict postoperative dysphagia [3, 10, 11, 16]. However, there are no clear clinical criteria about which parameter can be used effectively for predicting postoperative dysphagia, especially in patients with C2–3 KFS.

This study reported the influence of C2–3 KFS on preoperative and postoperative occipitocervical measurement parameters while undergoing OCF surgery. Despite the low incidence of KFS, forty patients in this study who underwent OCF surgery had C2–3 KFS. It seems that KFS is a common comorbidity for patients who are required to receive OCF surgery. In our study, we found that C2–3 KFS should not be an independent risk factor for dysphagia; however, C2–3 KFS will significantly interfere with the precise measurement of some common dysphagia-related occipitocervical parameters, such as the O-C2a and O-EAa. Due to the presence of bone deformity of the C2 vertebral body, especially vertebral body fusion in some patients with C2–3 KFS, it may be quite difficult to determine the inferior endplate of the C2 vertebra. There may be inaccurate measurements of the O-C2a and O-EAa.

Miyata et al. [13] reported that no postoperative dyspnea or dysphagia was observed in patients with positive dOC2a and recommend O-C2a should be equal to or slightly larger than the preoperative O-C2a during OCF. Ota et al. [15] demonstrated that a decrease in the O-C2 angle by 10° caused a reduction of the nPAS in the neutral position of approximately 37%. It means that the reduction of O-C2a has a significant effect on nPAS. Izeki et al. [14] revealed that the nPAS and postoperative dyspnea and/or dysphagia did not change over time once the O-C2a had been established after OCF. Hong et al. [6] reported a patient presented with dysphagia after OCF and the video-fluoroscopic study with barium showed nasopharyngeal regurgitation and delayed aspiration. They noted that hyperflexion of the upper cervical spine decreased the occipitocervical angle, which resulted in narrowing of the oropharyngeal space. The patient’s dysphagia resolved after revision operation. In this study, post-operative nPAS dramatically decreased in dysphagia group. 10 patients still experienced different level of dysphagia at final follow-up and no patients desired revision surgery because of improved neurological function. The cause of dysphagia after OCF was mainly mechanical narrowing of the oropharyngeal airway space regardless of inflammation, edema or neuropraxia, so the possibility of spontaneous recovery was small [14]. In such case, we recommend to perform early revision surgery to resolve dysphagia.

The simulation study combined with case-control study by Meng et al. [3] indicated that a dO-C2a of − 5° could be the threshold between dysphagia and normal swallowing. Wang et al. [11] demonstrated that the O-C2a could be critical predictor for postoperative dysphagia in patients undergoing OCF and avoiding O-C2a reduction greater than 5° could effectively avert postoperative dysphagia. Shimizu et al. [28] explained the risk of dysphagia and hyperlordotic/kyphotic malalignment secondary to excessive O-C2a decrease or increase and suggested that a change in O-C2a of > ± 5° would confer a risk of postoperative dysphagia and distal junctional kyphosis. O-C3a was measured on lateral x-rays in this study due to the difficulty in identifying the inferior endplate of the C2 vertebra caused by C2–3 vertebra fusion. O-C3a in the neutral position ranged from approximately − 16° to 29° in our patients. We discovered that using specific value of O-C3a directly had no predictive effect on postoperative dysphagia, similar to the O-C2a, but the dO-C3a did have a predictive effect on postoperative dysphagia. Therefore, we inferred that, due to the large variation in the cervical vertebra, there was poor accuracy when using only a specific value of the O-C3a to predict dysphagia.

The scatter diagrams showed that the dO-C3a, dO-C2a, dO-Da, and dOc-Axa were all linearly dependent on dnPAS in patients with C2–3 KFS, while there was a higher R^2^ value between the dO-C3a and dnPAS compared to those of dO-C2a, dO-Da and dOc-Axa. In the multiple regression analysis, it demonstrated that dO-C3a was the only significant parameter that affected the variation of nPAS. It meant that decrease of O-C3a may lead to decrease of nPAS which could pose a threat to the patient’s swallowing function after surgery.

Additionally, dO-C3a was significantly accurate as predictor for postoperative dysphagia in C2–3 KFS patients undergoing OCF surgery and the AUC of dO-C3a was the largest compared to dO-C2a, dO-Da and dOc-Axa. We hypothesized that the measurement error of O-C2a caused by difficulty in selecting the inferior endplate of C2 vertebrae due to C2–3 vertebral body fusion had an effect on the predictive ability of O-C2a for postoperative dysphagia. Therefore, the dO-C3a had a greater ability to predict postoperative dysphagia in patients with C2–3 KFS. Compared to conventional predictive value of postoperative dysphagia of a dO-C2a of − 5°, O-C3a reduction greater than 2° is a risky factor for predicting postoperative dysphagia. Because of more cervical segment involved when measuring O-C3a, a small angular change would result in a remarkable decrease in nPAS, which would further cause postoperative dysphagia. Based on our data and analysis, we recommended that the dO-C3a in OCF should be over − 2° in the neutral position to prevent dysphagia.

Nagashima et al. [20] proposed the Oc-Axa, a method of measuring the occipitocervical angle, particularly applicable to OCF with abnormalities of the C2 vertebral body. However, previous studies did not explore the strong relationship between the Oc-Axa and postoperative dysphagia after OCF. In addition, when measuring Oc-Axa, we also found the cases of circular external occipital protuberance and there may be positioning errors in the identification of the landmark, as pointed out by Nagashima. In this study, the obstacle to accurately measuring the O-Da and Oc-Axa was the abnormal morphology of the odontoid process in some patients, such as destruction, fracture, and/or deformation. For these patients, to decrease the measuring error, we selected the posterior longitudinal line of the C2 vertebral base to measure the O-Da and Oc-Axa. Despite the use of these methods, the measurement of O-C3a in C2–3 KFS patients could yield more accurate results than the measurement of O-Da and Oc-Axa.

There are several limitations in our present study. First, the study was a retrospective study. Second, when the odontoid process is deformed, the accurate measurement of the above angles will be affected to a certain extent. Third, we relied on patient reported symptoms of dysphagia without formal swallow evaluation like fiberoptic esophagoscopy or Dysphagia Short Questionnaire [29] pre- and post-operatively. In the end, the cohort in this study was relatively small and from a single patient population. A larger multi-institutional study should be conducted to more accurately predict postoperative dysphagia with appropriate parameters after OCF surgery especially in patients with C2–3 KFS.

## Conclusion

Measurement of the O-C2a and O-EAa may not be reliable for patients with C2–3 KFS due to the unclear inferior endplate of the C2 vertebra. In such case, the dO-C3a is the most reliable measurement indicator for evaluating occipitospinal alignment and predicting postoperative dysphagia in patients undergoing OCF. Moreover, the dO-C3a should be no less than − 2° during OCF surgery to reduce occurrence of postoperative dysphagia.

## Data Availability

The datasets used and analyzed during the current study are not publicly available due to personal information security and privacy protection but are available from the corresponding author on reasonable request.

## Abbreviations

OCF: Occipitocervical fusion
O-C2a: Occipital to C2 angle
O-EAa: Occipital and external acoustic meatus to axis angle
KFS: Klippel-Feil syndrome
O-C3a: Occipital to C3 angle
O-Da: Occipito-odontoid angle
Oc-Axa: Occipital to axial angle
nPAS: Narrowest oropharyngeal airway space
ROC: Receiver-operating characteristic
AUC: Area under the curve
BI: Basilar invagination
AS: Atlantoaxial subluxation
RA: Rheumatoid arthritis
ARS: Anterior release surgery
CI: Confidence interval
